# Case in Which the Urachus Remained Open, and a Ring-shaped Calculus, Formed on a Curved Hair in the Bladder, Was Extracted through the Umbilicus

**Published:** 1850-11

**Authors:** Thomas Paget


*Case in which the Urachus remained open, and a ring-shaped Calculus, formed on a curved hair in the Bladder, was extracted through the Umbilicus.* By Thomas Paget, Esq.—[In our number of January, 1848, p. 313, will be found recorded, by Dr. R. G. Cabell, of Virginia, a case of pervious urachus in an adult. Another example of this rare malformation has recently (June 11) been communicated to the Royal Medical and Chirurgical Society, by Mr. Paget.]—*Am. Jour. Med. Sci.*

J. C., aged forty years, had suffered for more than a year from frequent and painful micturition. On sounding, a calculus was readily detected. He mentioned, that upon attempting to make water, and during violent efforts at work, a portion of the urine sometimes escapes at the navel, which is open, and that, so far as he knows, it has been so from his birth. The catheter introduced into the bladder through the urethra was easily made to appear at the umbilical opening. Thinking that the stone might be extracted through this aperture, Mr. Paget distended the bladder to the utmost with warm water, the umbilical aperture being tightly plugged, the patient reclining with his head lower than the pelvis, and with the intention of removing the plug, thinking that the calculus might be flushed out with the water; but it then occurred to him to try first the finger at the umbilicus. It readily passed, and when at full length down the unnatural passage, caught within the circle of the calculus sufficiently to enable him to drag it to the side of the bladder and extract it. The nucleus was found to be a hair, and on carefully truncating it, the projecting extremity was seen. The phenomena connected with the opening are thus described: There is a circular deficiency in the linea alba, an inch in diameter, its margin being thickened, and of cartilaginous hardness. Through this protrudes a hernia of the size of a goose-egg, which, in lieu of ordinary integument, is covered by mucous membrane, the surface, however, becoming dry, when exposed for any length of time. He never makes water whilst the hernia is out, for when called to an effort for that purpose, the first act of the bladder is gradually to draw into the abdomen the whole of the protruded substance; its first contractions have no other effect, and it seems not to have the power to force the urethra until that is accomplished. At the latter part of this act, at the instant of the disappearance of the hernia, there occurs rather a forcible jet of urine from the opening; the flow from the urethra also commences at this juncture, and the bladder is emptied in the usual way, the jet from the umbilicus ceasing, not to be renewed, except by a violent accelerating action of the expulsor muscles. He can retain a pint of urine. By watching the first contractions of the bladder, it becomes evident that to the thickened margin of the umbilical aperture are attached the muscular fibres of the bladder extended along the urachus. The pouch of the hernia is formed by eversion of the posterior part of the neck only, which is of course attached to the upper half of the aperture, and when protruded presses upon the hard edge of the lower half sufficiently to prevent the escape of the urine, except under restraining efforts of the abdominal muscles. He wears a girdle, with a thick pad of flannel to catch the urine. With the extraction of the calculus, all the bladder symptoms ceased, and it was thought unadvisable to interfere with the congenital defect.

